# Comparative Analysis of Thermophysiological Comfort-Related Properties of Elastic Knitted Fabrics for Cycling Sportswear

**DOI:** 10.3390/ma13184024

**Published:** 2020-09-10

**Authors:** Yetanawork Teyeme, Benny Malengier, Tamrat Tesfaye, Simona Vasile, Lieva Van Langenhove

**Affiliations:** 1 Department of Materials, Textiles and Chemical Engineering, Faculty of Engineering and Architecture, Ghent University, B-9052 Ghent, Belgium; Benny.Malengier@UGent.be (B.M.); Lieva.VanLangenhove@UGent.be (L.V.L.); 2 Ethiopian Institute of Textile and Fashion Technology (EiTEX), Bahir Dar University, 6000 Bahir Dar, Ethiopia; tamrat_tsfy@yahoo.com; 3 Department of Fashion, Textiles and Wood Technology, University College Ghent, B-9000 Ghent, Belgium; simona.vasile@hogent.be

**Keywords:** knitted fabric, thermal conductivity, moisture management, air permeability, water vapor permeability, drying time

## Abstract

This research focused on the investigation of the thermophysiological comfort properties of four selected knitted fabrics of different fiber blend ratios suitable for cycling wear. Comfort-related properties of the fabrics were determined and compared including air permeability, moisture management properties, drying time, thermal conductivity, and water vapor permeability. For those comfort properties of the fabric to be correlated, fabric structural properties, fabric density, fabric weight, and fabric thickness have been considered. Suited fabrics should have good air permeability, thermal conductivity, moisture management properties, and a short drying time. According to the measurement results, the fabric polyamide/elasane (58/42 PA6.6/EL) with good air permeability, thermal conductivity, moisture management properties, and short drying time was more suited for summer cycling clothing. Furthermore, this paper provides a new understanding of considerations that are needed for several end uses involving specific activity levels.

## 1. Introduction

Over the last few years, sales of casual and sports clothing have been growing all over the world, so manufacturers and researchers have devoted studies to this field [1,2,3,4,5]. While fashion is the key element in selecting daily clothing, clothing comfort has become a crucial parameter for sports clothing [6,7]. It influences the performance and efficiency of the wearer as well. Wear comfort is a complex phenomenon, and thermophysiological comfort is significantly important for athletic apparel and next-to-skin applications as it directly impacts an individual’s skin [8,9,10,11]. It incorporates heat and moisture transport through clothing, and key notions comprise thermal insulation, breathability, and moisture management [12].

As a result of the significant level of metabolic heat generation, which is in the range of 800–1300 W for high-activity sports, heat stress is of great concern [13,14,15]. Furthermore, moisture is extensively known as the most important factor contributing to discomfort sensations [16,17,18]. Hence, sweat absorbing, fast drying, and cooling are the key functional requirements for high-activity sports clothing [19]. Moreover, high stretch and recovery to provide the appropriate fit and allow freedom of movement to the wearer is required [12].

Cycling is the most popular sport all over the world [20,21]. It is a long-duration sport that can be performed in different climate conditions; in this manner, in every period, cyclists can use a collection of garments. Besides, cycling is not quite the same as different sports and has numerous features, for example, high energy utilization, high perspiration rate, and a higher limb exercise and range of activity to the lower body compared to the upper body [20,21,22]. Therefore, as indicated by the attributes of cycling sports, the fabrics used for cycling should be functional and specifically designed to meet the athlete’s requirements.

Cycling jerseys should adjust to the movement of the body to stretch and make human body motion attractive under the state of satisfying the reason of the garments’ aesthetic impact. Above all, it ought to have different features, for example, a proficient component to deal with sweat, a high dampness permeability, heat conservation, windproof execution, protection from sunlight, and superb washing performance [23].

Many studies have been carried out on wear comfort in relation to the type of yarn, fabric construction parameters, fabric finishing, etc. For instance, Bhattacharya and Ajmeri compared the knitted fabric structures produced from viscose and the excel yarn porosity and evaluated the air permeability property of sports clothing. They declared that because of their higher air permeability, excel single jersey fabrics are considered ideal candidates for hot-weather sportswear [24]. Öner and Okur considered two microclimate conditions: One where wetness is not felt by the body and sweating occurs through evaporation, and the other where body temperature is raised quickly, and wetness occurs during sports activities. They showed that the comfort parameters in the wet state and dry state of fabric are different [25]. Baker et al. designed and evaluated a single-layer garment that incorporates different textiles to improve the physiological comfort of backpackers hiking in cold weather conditions using a combination of three fabrics, specifically a thermal fabric, a moisture-wicking fabric, and a control fabric, strategically placed in different areas of the body. The backpacker was satisfied by the body heat retention and dissipation characteristics of the specialized fabrics in targeted body areas [26].

In Bedek et al., six types of knitted underwear products were analyzed in terms of the textile and thermal comfort properties. Correlations were found between the vapor resistance and the fabric thickness, moisture retention, and drying time [27]. In a comparative study, Frydrych found that fabrics made of cotton yarn have better thermal properties than those of fabric made of Tencel [28]. Yanılmaz and Kalaoglu studied the relationship between various knitted fabric structures and some parameters of thermophysiological comfort. The authors stated that the structure of knitted fabrics had a significant influence on transfer wicking ratios, wicking height, contact angle values, and water evaporation rate [29]. Raja and his colleagues investigated single-jersey knitted fabrics manufactured from ring, compact, and ring/compact folded yarns in terms of thermal properties. They found that the thermal properties of single-jersey folded yarn fabric is affected by the twist of the yarn and traveler weight [30]. Vasile et al. investigated the Fabric Touch Tester (FTT) device-predicted primary comfort indices (i.e., smoothness, softness, and warmth) of polyester-cotton knitted fabrics and compared them to finished knitted fabrics (i.e., dyed and dyed with softening treatments). Regardless of the type of yarn, it was found that significant differences exist between the comfort indices of the untreated fabrics and the finished fabrics [31].

Characterization and analysis of textile fabrics to assess their comfort properties for cycling clothes are the first step toward their application. To determine their comfort properties for these applications, it is important to understand the thermal and sensorial fabric properties. Studies have been done in this area, though the studies were focused on specific applications, e.g., design materials precisely adapted to a given activity [27,32]; investigating the effect of yarn twist, knitted structures, and type of raw material on thermal property variations of finished goods [33,34,35]; designing backpacker’s hiking garments in cold weather conditions, and determination of the overall comfort performance for underwear fabrics [28,29].

The purpose of this study was to compare the thermophysiological comfort properties of double tricot, single tricot, and tricot with pillar stitch polyamides knitted fabrics, which is determined to be advantageous in future studies by the authors, and to design different sports cycling wear by using these fabric types. In addition to the variation in research findings of the thermal comfort properties of the elastic knitted fabric, we proposed a set of tests for companies to support the quality control and selection of fabrics for the same purpose, to provide a well-informed platform in the design and development of future cycling functional garments.

## 2. Materials and Methods

### 2.1. Materials

Four different types of knitted fabrics denoted as A, B, C, and D were selected among the most commonly used fabrics for cycling wear and were tested using textile-based laboratory methods. Material composition, fabric weight, thickness, density, porosity, elongation, and stitch density of fabrics A–D are indicated in Table 1. Material composition and elongation information were obtained from the fabric manufacturer. The authors of the work obtained all the other data. Their mass per unit area varies largely between 146 (fabric A) and 265 g/m^2^ (fabric D) and they contain blends of PA6.6 with a variable amount of elastane between 25% (fabric C) and 43% (fabric D). Fabrics B and C have a similar structure, warp knit (Double tricot); fabric A has a single tricot; and fabric D has a tricot with pillar stitch knit structure. This fabric D exhibits the lowest porosity (45.5%), while fabrics B and C have the highest (around 62%). Although all materials are aimed toward cyclist garments, a large difference in properties can be observed. A thinner fabric can be used, but a lower porosity is then used to arrive at a higher bulk density, while a heavier fabric can be created through a different knitting technique for the same thickness.

### 2.2. Methods

#### 2.2.1. Sample Preparation

The samples were conditioned at 65% ± 4% relative humidity and 21 °C ± 2 °C temperature for at least 24 h in a controlled conditioning chamber according to EN-ISO 137:2005 [36]. Afterward, the samples were characterized for their thermophysiological comfort properties including air permeability, moisture management, water vapor permeability, drying time, and thermal properties.

#### 2.2.2. Thickness Measurement

The thickness of the sample fabrics were measured according to ASTM D1777:2007 [37] using a digital fabric thickness tester.

#### 2.2.3. Bulk Density

Bulk density of the fabrics (kg·m^−3^) was calculated as the ratio of fabric mass per unit area (g·m^−2^) and thickness (mm).

#### 2.2.4. Porosity

Relative porosity P (%) was computed using Equation (1):

(1) $$P=\left(1-\frac{m}{\rho \times d}\right)\times 100$$

where *m* is fabric mass per unit area (g·m^−2^), *ρ* is the fiber density (g·m^−3^), and *d* is the fabric thickness (m).

#### 2.2.5. Air Permeability

Air permeability properties of the fabrics (mm/s) were measured using an air permeability instrument (EMI Development, Bréviandes, France), at 100 Pa air pressure and a 20 cm^2^ test area according ISO 9237:1995 [38]. The results are the average of ten measurements taken at random in various regions of the fabric.

#### 2.2.6. Moisture Management

Moisture management properties were assessed by a Moisture Management Tester (MMT, SDL Atlas, Rock Hill, SC, USA) according to AATCC 195: 2011 [39]. The MMT provides a complete profile of a fabrics’ (particularly fabrics worn next to the skin) performance by measuring wetting time (WT), maximum wetted radius (MWR), spreading speed (SS), and absorption rate (AR) on the top surface (T) and bottom surface (B) of the fabrics, and by calculating the accumulative one-way transport capability (R) and Overall Moisture Management Capacity (OMMC), which has values in the interval 0–1. Based on these parameters, the MMT distinguishes seven fabric categories: Waterproof fabric; fast-absorbing and quick-drying fabric; slow-absorbing and slow-drying fabric; water repellent fabric; fast-absorbing and slow-drying fabric; water penetration fabric; and finally, moisture management fabric. A grading 1–5 (low–high) is applied to all indices. Ten specimens of 8 cm × 8 cm were tested, and the average was calculated.

#### 2.2.7. Water Vapor Permeability

Water vapor permeability WVP (g/m^2^·Pa·h) or breathability describes the amount of water vapor diffusing through the textiles per square meter, per hour, and per unit of water vapor pressure difference across the textile. The inverted cup method was used to test the specimen’s water vapor permeability WVP, which was then calculated according to ISO 15496:2015 [40]. For each fabric quality, three circular test specimens (diameter 110 mm) were tested and the mean WVP was calculated.

#### 2.2.8. Drying Time

Drying time was determined according to ISO 17617:2014 [41] vertical method A2 with the fabric placed on a top-pan balance and the specimen exposed to the test environment from both sides. A specified quantity of moisture was applied in the middle of a specimen and the weight of moisture remaining on the specimen after a specific period was measured. Drying rate DR (%/min) is defined as the length of time required to dry a known mass of moisture from textile fabric. Drying time is the time (min) for which 100% of the water loss occurs. Two perspiration solutions (alkaline pH 5.5 and acid pH 8.03) were prepared according to ISO 105-E04:2013 [42]. Three square test specimens (100 × 100 mm) were prepared and conditioned as mentioned in Section 2.2.1 for each fabric quality. A quantity of 0.08 ± 0.01 mL of conditioned (24 h at 20 ± 2 °C) artificial perspiration was applied in the center of the exposed upper surface (i.e., inside of the fabric, which comes in contact with the skin) with a micropipette. The initial weight of the sample (M_0_) was recorded and the weight was then measured at 5 min intervals, for a total test period of 60 min. Some deviations from the testing norm apply: A hanging frame holding the specimen was kept in the climatic chamber (65% RH, 21 °C) and its weight was measured every 5 min, with a balance with 0.001 mg precision, which was placed inside a closed chamber to keep the air velocity less than 0.1 m/s as required.

#### 2.2.9. Thermal Properties

To assess the thermal properties of the fabrics, a Fabric Touch Tester (FTT, SDL Atlas, Rock Hill, SC, USA) [43] was used. The FTT measures 13 fabric indices in total [44] related to compression, bending, surface friction and roughness, and thermal properties. In this study, measured fabric thermal indices (Qmax, TCC, TCR) were considered. Qmax (W·mm^−2^) is the maximum (thermal) energy transmitted during compression through the sample. Thermal conductivity (10^−3^ W·m^−1^ °C^−1^) represents the energy transmitted per degree per meter per second when the specimen is under compression (TCC) and under recovery (TCR) [45]. The FTT calculates active and passive warmth, which represent the warmth that would be perceived by humans when squeezing the fabric with fingers and when passively worn on the skin, respectively. No testing standard currently exists; therefore, the fabrics were tested according to the testing protocol of the equipment manufacturer. For each fabric quality, a few tens of specimens (L-Shape of 28 cm) were tested. The results presented are the average of ten measurements (5 specimens for fabric outside and 5 specimens for inside).

## 3. Results and Discussion

Results of air permeability, moisture management properties, drying time, thermal conductivity, and water vapor permeability of the knitted fabrics are presented below. The results of variance analyses of the observed measurements and the differences between each group have been explained using the *t*-test (alfa = 0.05) and Pearson correlation.

### 3.1. Air permeability

**Figure 1:**
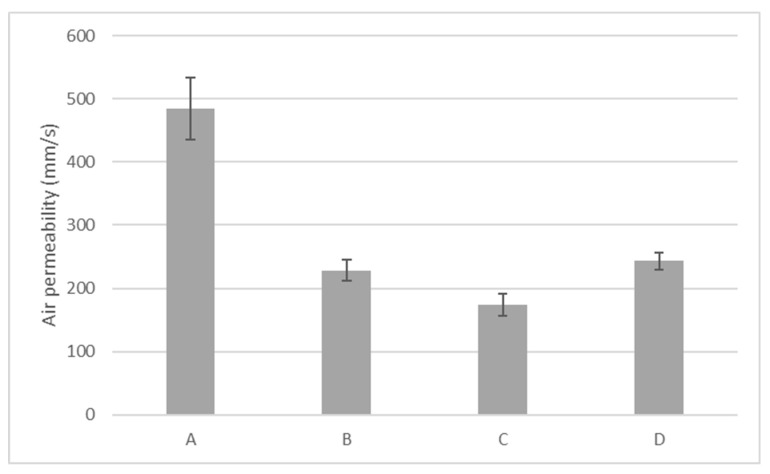
Air permeability (mm/s).

The data in Figure 1 illustrate the average air permeability of the tested samples. It was noted that fabrics B and C had (comparable) the lowest air permeability and fabric A had the highest air permeability value (485 mm/s). Air permeability was negatively correlated with fabric thickness (Pearson coefficient −0.86, significant at 0.01 level). Among the fabrics, fabric A had one of the lowest porosities (55.3%) and had the lowest thickness and highest bulk density (511 kg/cm^3^). Sample B and C with similar structure and comparable properties (Table 1) also had comparable air permeability. The tricot with pillar stitch samples (D) depict a lower air permeability than fabric A, and this could be due to its compact surface with more fiber content, highest bulk density (623 kg/cm^3^), and lowest porosity (45.5%). Therefore, from the result, it can be concluded that loosely knitted fabric structures with a higher amount of entrapped air exhibit a better air permeability property owing to their higher porosity. This is in agreement with work of Nazir et al. who found out that the interlock knitting of fabric at a lower gauge and higher stitch length results in a loose structure with larger air gaps as compared to the tighter fabric structure [46].

### 3.2. Moisture Management

The structural properties (i.e., fabric density, thickness, porosity, weight, …) and material type of fabrics are crucial in the determination of the moisture management properties of fabrics. The ultimate objective of managing moisture in fabrics is to ensure moisture is transported to the outer surface in the shortest possible time [47]. The knitting parameters (such as structure and stitch density) predominantly influence the knitted fabric physical characteristics (i.e., thickness, weight, and porosity), which thus influence the fabric comfort properties [48].

All the fabric samples were tested, and Table 2 summarizes the mean values (±SD) of all corresponding indices. Fabric A had the highest one-way transport capacity R (385%), indicating that liquid sweat can be easily and quickly transferred from the skin to the outer surface. This fabric also had a high spreading speed and low wetted radius for the inner and outer surfaces (8.33 mm), showing that liquid passed immediately through the fabric without wetting it. Overall, fabric D with a tricot with pillar stitch structure exhibited a different behavior and a large variability (SD) of OMMC among others, indicating that, depending on the location of the wetting, the fabric reacts quite differently. Unlike fabrics A–C, it showed a mean negative accumulative one-way transport index with slow transport of moisture to the outside (side B). This was the thickest fabric that also had a different structure than fabrics A–C. This could be due to its structural difference, which had a Tricot with pillar stitch structure, and the water droplet had a different behavior than on a smooth surface. The accumulative one-way transport index was positive and sometimes negative even if the direction of the pillar stich was the same. This could be due to the positioning of the pillar stich during testing, and the water droplet had another trajectory and sometimes went along the channels and remained on the side exposed to water. Further research is required to provide the possible reasoning for these characteristics. Fabric B had similar behavior with fabric D but exhibited less variability and a better one-way transport index. Unlike smooth fabrics A–C, fabric D had a tricot with pillar stitch structure, which may potentially lead to an irregular spreading pattern of the liquid moisture as demonstrated by the top/bottom wetted radius (min. 5 mm, max. 20 mm) and large variability of wetting time, absorption rate, and spreading speed. Consequently, it will lead to large variability (SD = 0.17) of OMMC (average 0.20), which is computed as the sum of one-way transport capability, spreading speed, and absorption time, where one-way transport capability has the highest weighting factor (0.5).

**Figure 2:**
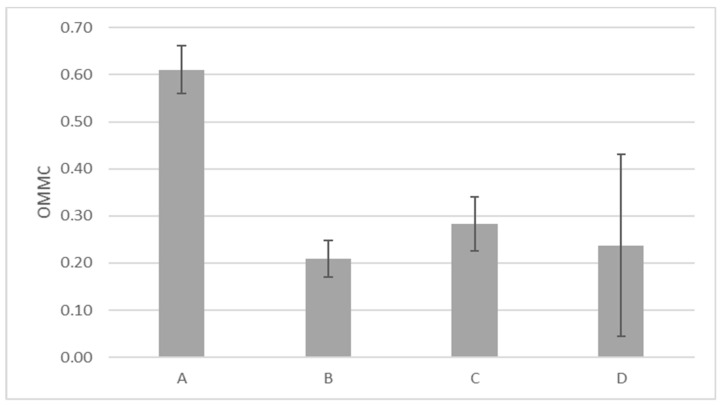
The test result of fabrics’ moisture management capability.

Figure 2 illustrates the OMMC of the tested fabrics. Three of the fabrics had average OMMC values between 0.2 and 0.3 and fabric A had the highest OMMC (0.61). As previously mentioned, OMMC considers the moisture absorption rate, spreading speed, and accumulative one-way transport of the liquid moisture from the outer to inner surface of the fabric. Fabrics A and C were classified as water penetration fabrics, fabrics B and D as fast-absorbing fabrics, and B as a fast-drying fabric. This means that fabric A and C exhibit a small spreading area of sweat and a means of excellent one-way transport. Some characteristics of fabrics B and D are an average to fast wetting and absorption, small spreading area, fast-spreading, and poor one-way transport. In a clothing system, it may be useful to alternate fast absorbent fabrics B/D with fabrics A/C that quickly transport sweat outside, depending on the quantity of sweat generated [49].

### 3.3. Water Vapor Permeability

**Figure 3:**
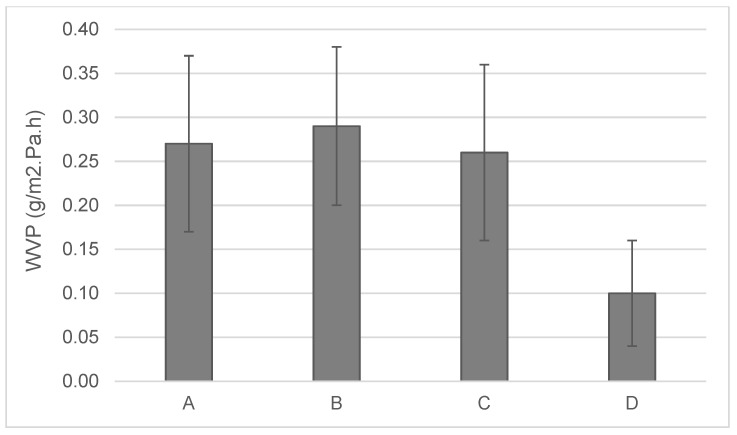
Illustration of the fabrics’ breathability (g/m^2^·Pa·h).

Water vapor transmission from the wearers’ body is predominantly important as it can effectively support in managing the thermal condition of the body [50]. Figure 3 shows the water vapor permeability (WVP) of the tested fabrics. With the water vapor permeability of the sample, fabric (B) had the highest breathability (0.29 g/m^2^·Pa·h) and fabric (D) was the least breathable (0.097 g/m^2^·Pa·h). The comfort of textile fabrics for the cyclist improved as the water vapor permeability increased, so based on the data in Figure 3, fabric B would be best for sweat vapor transport from the skin to outside. Moreover, fabric B is considered fast-absorbing and fast-drying as compared to the other fabrics. Hence, fabric B shows a potentially good comfort property for cyclists.

### 3.4. Drying Time

**Figure 4:**
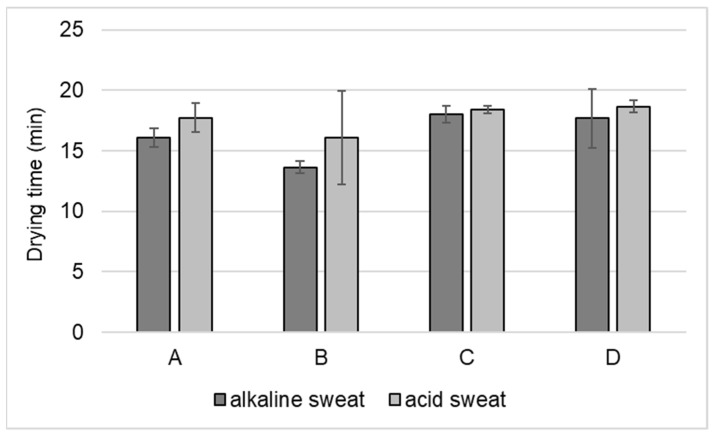
Drying time (100%) of the fabrics with alkaline and acid artificial perspiration.

The average fabrics’ drying time is shown in Figure 4 depending on the type of artificial perspiration used. All fabrics had a drying time between 16 and 18.7 min with fabric B drying fastest (13.6 min) and fabric D drying slowest (18.7 min, for alkaline sweat). In general, low variances were observed for the drying time of most fabrics, as shown by the standard deviation bars, except for fabric B and D in the case of acid perspiration. Differences can be observed between the two types of perspirations, and acid sweat seems to lead to a longer drying time in all cases but with no statistically significant differences (*t*-test, alpha = 0.05, *p* > 0.05). This difference could be due to the difference in porosity, construction, weight of the fabric, and effect of chemicals on fiber properties as this determines the water trapped inside the fiber.

### 3.5. Thermal Properties

**Figure 5:**
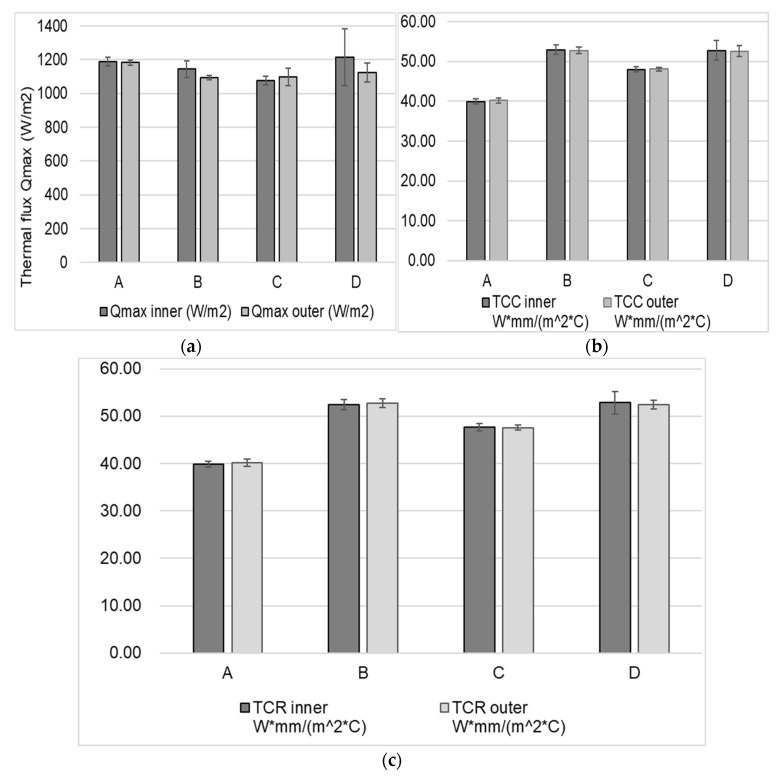
Thermal flux Qmax (**a**), and thermal conductivity during compression TCC (**b**) and recovery TCR (**c**) for the inner and outer side of the fabrics.

The properties of thermal comfort for the tested samples are illustrated in Figure 5. Measured values of Qmax (maximum thermal flux measured that passed through the fabric) and thermal conductivity during recovery (TCR) are shown in Figure 5a,b, respectively, both for the inner and outer side of the fabrics. Some differences can be observed for the maximum thermal flux that passed through the fabric, depending on the testing position of the inner/outer side of fabrics B and D. This could be due to the different fabric structure exhibiting different characteristics in the face and back side of the fabric. As expected, no difference exists between the thermal conductivity TCR of the fabrics depending on the testing position of the inner/outer side.

Both thermal conductivity during compression (TCC inner/outer) and thermal conductivity during recovery (TCR inner/outer) were correlated with mass per unit area (Pearson coefficient of 0.55/0.54, respectively, significant at 0.05/0.01 level; and 0.59, respectively, with significance level of 0.01). Similarly, the thickness was correlated with TCC inner/outer side (all Pearson coefficients around 0.90, significance level 0.01). For example, the thickest/heaviest fabric D (0.44 mm/265 g·m^−2^) exhibited the highest thermal conductivity during compression (TCC) and recovery (TCR), and the thinnest/lightest fabric A (0.29 mm/146 g·m^−2^) was registered with the lowest values of thermal conductivity during compression (TCC) and recovery (TCR), as shown in Figure 5b,c. Among the fabrics considered, the results suggest that fabrics B and D will perform better in heat transport, potentially ensuring better comfort in the case of intense activity when a low amount of energy must be disposed to ensure thermal comfort. This agrees with previous studies and could be due to the thickness and porosity of the fabric, as it provides higher thermal insulation (Tabor, 2020) [49].

## 4. Conclusions

This paper presented a set of test methods to quantitatively evaluate stretch-polyamide knitted fabrics commonly used for cycling outfits. Variable behavior with respect to air, water, vapor, and heat management was noticed, which suggests different thermophysiological comforts of the wearer depending on the fabric used. Among the fabric considered, fabrics B and D seemed to be a better candidate in heat transport, potentially ensuring better comfort in the case of intense activity when a low amount of energy must be disposed to ensure thermal comfort. On the other hand, fabric A was most permeable to air and had the best liquid moisture management. Fabric B, which dried fastest (13.6 min) than all the fabrics, exhibited a moisture drying time between 16 and 18.7 min for alkaline sweat. Some difference was observed between the two types of sweat, with acid sweat leading to a longer drying time, in most of the cases. In general, according to test results, the fabric (58/42 PA6.6/EL) with good air permeability (485 mm/s), moisture management properties (0.61), and short drying time (about 16 min) was more suited for summer cycling clothing. These sets of tests can be employed mostly for quality control, to support companies in the comparison and selection of fabrics for the same purpose. The tests were performed in lab conditions, on single-layer fabrics, and they only indicated the potential of these fabrics to ensure good thermophysiological comfort of the cyclists. In real scenarios, additional factors like garment fit, cycling intensity, and environmental conditions are of uttermost importance contribute to the comfort sensation of the wearer. Future studies will entail on the effects of these fabrics while used as single-layer tight-fitted garments, and identification of the basic mechanisms is essential in order to measure their function as either a performance aid or support tool for athletes during both training and competition.

## Figures and Tables

**Table 1:** Mean values (SD) for the structural parameters of fabrics A–D.

Sample Code	Raw Material % (PA6.6/EL)	Structure	Mass per Unit Area (g/m^2^)	Thickness (mm)	Bulk Density (kg/cm^3^)	Porosity %	Stitch Density		Elongation %	
							Wales/cm	Course/cm	L	W
A	58/42	Single tricot	146.4 (5.85)	0.29 (0.01)	511.82 (8.03)	55.3 (0.7)	31	37	125	157
B	64/36	Double tricot	179.64 (3.39)	0.42 (0.00)	427.71 (8.06)	62.6 (0.7)	32	39	160	130
C	75/25	Double tricot	172.82 (0.66)	0.40 (0.00)	434.27 (5.46)	62 (0.5)	31	48	135	185
D	57/43	Tricot with pillar stitch	273.06 (4.46)	0.44 (0.01)	623.51 (10.04)	45.5 (0.9)	19	29	155	170

PA6.6—polyamide 6.6; EL—elastane.

**Table 2:** Mean values (±SD) of moisture management parameters assessed by Moisture Management Tester (MMT) for fabrics A–D.

Sample Code	Side	Wetting Time (s)	Absorption Rate (%/s)	Max Wetted Radius (mm)	Spreading Speed mm/s	Accumulative One-Way Transport Index R (%)	OMMC
A	T	5.73 (0.4)	50.19 (32.35)	8.33 (2.46)	0.89 (0.08)	385.76 (52.7)	0.61 (0.05)
	B	9.59 (2.6)	60.42 (8.29)	8.33 (2.46)	0.59 (0.17)		
B	T	6.40 (0.5)	42.17 (18.1)	10.0 (0)	0.83 (0.06)	125.80 (33.7)	0.2 (0.03)
	B	20.04 (5.8)	14.87 (1)	10.0 (0)	0.37 (0.13)		
C	T	7.24 (0.7)	53.23 (7.1)	5.0 (0)	0.68 (0.08)	145.91 (38.0)	0.28 (0.05)
	B	18.82 (2.2)	33.60 (6.5)	5.0 (0)	0.27 (0.03)		
D	T	7.31 (1.8)	46.56 (20.18)	11.5 (4)	0.84 (0.25)	–46.47 (280.36)	0.20 (0.17)
	B	14.21 (7.48)	33.46 (19.60)	12.25 (3.8)	0.57 (0.19)		

T—fabric top (inside, in contact with skin); B—fabric bottom (outside).
